# Treatment of Eccrine Carcinoma of the Chin via Submental Island Flap

**Published:** 2010-04-08

**Authors:** Effie Pappas-Politis, David C. Driscoll, Yvonne N. Pierpont, Paul R. Albear, William L. Carter, Lisa J. Gould

**Affiliations:** ^a^Division of Plastic Surgery, University of South Florida, Tampa; ^b^Institute for Tissue Regeneration, Repair, & Rehabilitation, Bay Pines VA Healthcare System, Bay Pines, Fla; ^c^Section of Plastic Surgery, James A. Haley Veterans' Hospital, Tampa, Fla

## Abstract

**Background:** The submental artery island flap is a reliable reconstructive option for lower face defects. Advantages of this flap include suppleness of tissue, excellent color and texture match to facial skin, a wide arc of rotation, prominent blood supply, and a well-hidden donor site scar. **Methods:** This article describes a 61-year-old man with eccrine carcinoma of the chin necessitating extensive excision. A submental artery island flap was used for the reconstruction of the extended chin subunit. **Results:** The operation resulted in excellent aesthetic outcome and maintenance of oral competence. **Conclusion:** The submental artery island flap utilizes loose tissue of the submental area to effectively and reliably reconstruct the soft tissue subunits of the chin and is a superb option for sizable defects. The flap is sufficiently well vascularized to tolerate postoperative radiation therapy without significant fibrosis or retraction.

The chin is an aesthetically and functionally important anatomic subunit of the face, bordered by the lower lip superiorly and the mandible/submandibular line inferiorly. The soft tissue formation of the chin includes the vermilion component of the lip, multiple muscle layers, and the gonion with firm muscular and boney attachments.^1^^-^4 Soft tissue reconstruction in this area involves the challenge of maintaining normal function and aesthetics of the lower lip, mentum, and neck. In addition, the relative paucity of regional tissues available makes reconstruction of this area difficult. In order to reconstruct the entire chin subunit, the submental island flap utilizes the loose soft tissue of the submental area, while maintaining the aesthetic subunits of the chin, thus preventing lower lip retraction and allowing reconstruction and maintenance of the submental aesthetic contour.^1^^-^6 In this report, we present the use of the submental artery flap for reconstruction of an extended chin subunit.

## CASE REPORT

**Figure 1:**
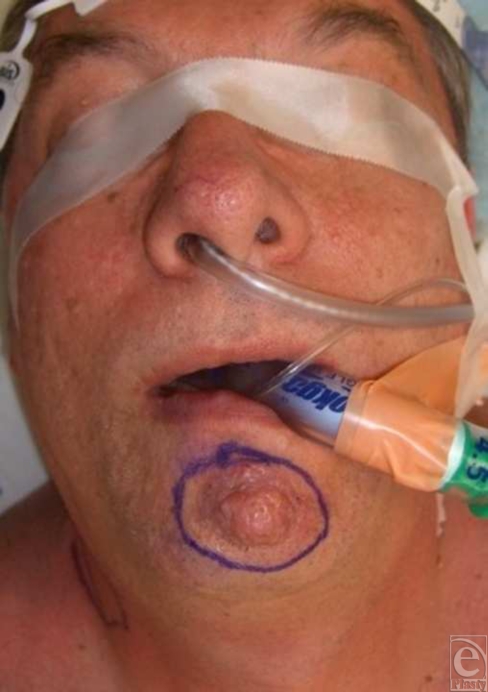
Preoperative view of eccrine carcinoma lesion of the chin.

A 61-year-old man presented with a large exophytic lesion of the chin, which had been present for 10 years with recent enlargement over the previous 6 months (Fig 1). He denied pain or bleeding, but recently noted some ulceration in the central portion of the lesion. Physical examination showed a 1.5 × 1.0 cm mobile lesion centrally located at the mental crease of the chin. There were no notable sensory or motor nerve abnormalities and no palpable lymphadenopathy of the submental, submandibular, anterior, or posterior triangles of the neck. Intraoral examination was normal. Biopsy of the lesion demonstrated eccrine carcinoma. Computed tomography imaging of the neck showed no evidence of metastatic disease.

**Figure 2:**
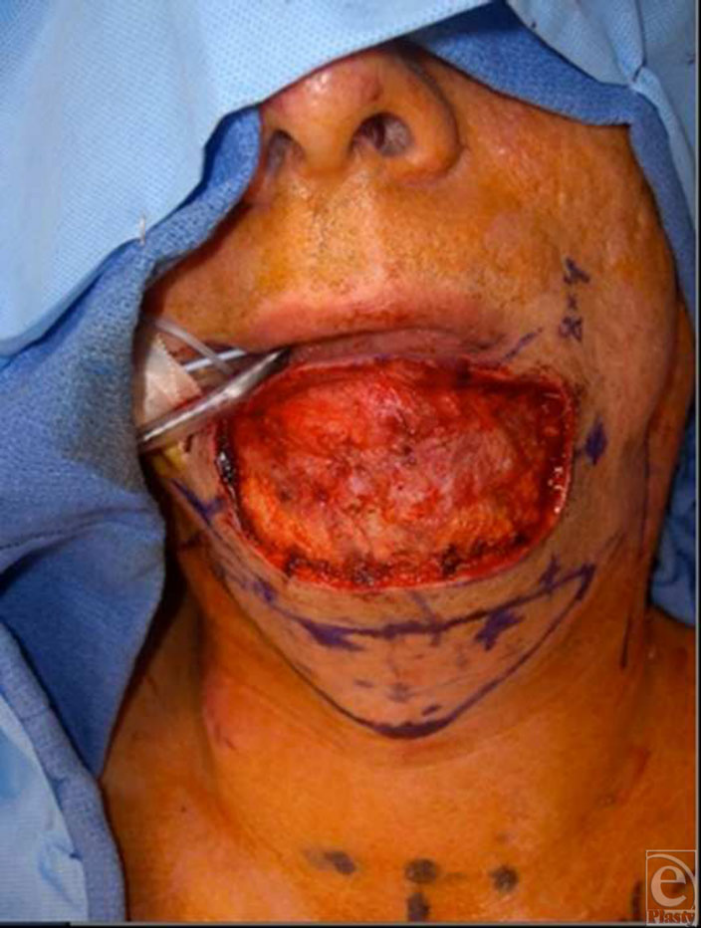
Intraoperative view of chin defect and submental artery island flap design.

Treatment plan involved initial wide excision of the lesion and surrounding borders, including skin and subcutaneous tissue. The defect was closed using a full thickness skin graft from his right neck. Permanent pathology demonstrated eccrine carinoma with positive deep and peripheral margins including platysma muscle invasion. Reexcision involved wider and deeper margins, which were inclusive of the entire chin subunit up to the vermilion border. Frozen sections done at the time of surgery were negative. The total defect measured 9 × 5 cm (Fig 2).

**Figure 3:**
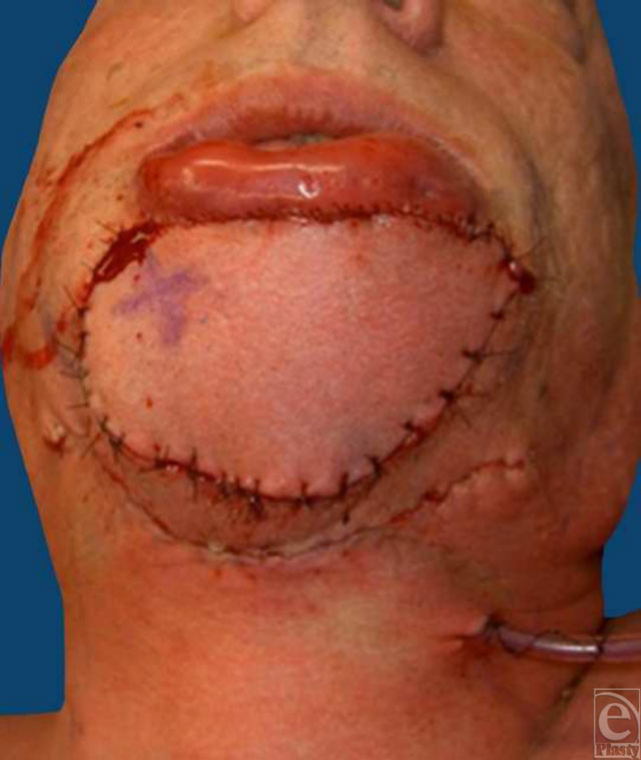
Intraoperative view of extended chin reconstruction with submental artery island flap inset.

The submental artery island flap design for defect coverage utilized a fusiform-shaped flap with the superior aspect of the flap beginning 1.5 cm inferior to the bony mandibular margin of the defect, and lateral edges extending to the approximate level of the mandibular angle bilaterally. The total flap skin paddle measured 13 × 6 cm. The submental artery was identified bilaterally just inferior to the mandibular angle. Dissection of the flap proceeded in the subplatysmal plane and right and left pedicles were identified. Marginal mandibular nerves were identified and preserved. The left pedicle was ligated to allow inset of the flap as an island based on the right-sided perforator. Subsequent to passing under a skin bridge between the defect and flap donor site, the perforator artery flap was trimmed, contoured, and inset into the chin defect (Fig 3). The donor defect was closed primarily after undermining of the surrounding skin. Postoperative course was uncomplicated aside from swelling of the lip and surgical site. The flap was well perfused with no evidence of vascular compromise, and the swelling gradually decreased.

**Figure 4:**
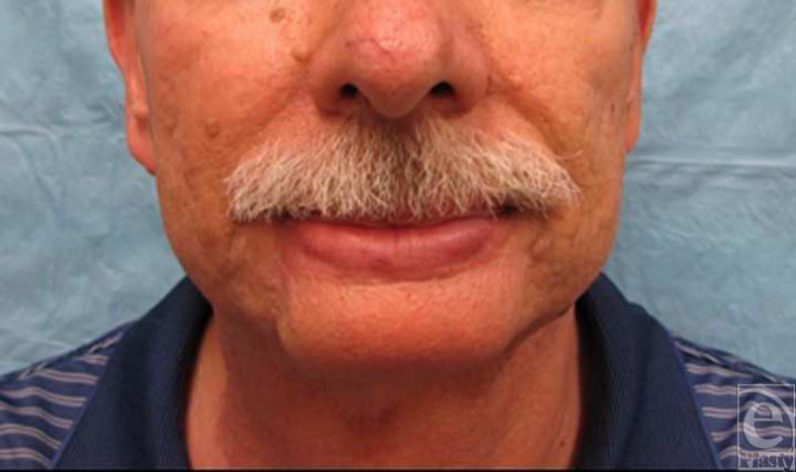
One-year postoperative result of extended chin reconstruction with submental artery island flap—frontal view.

**Figure 5:**
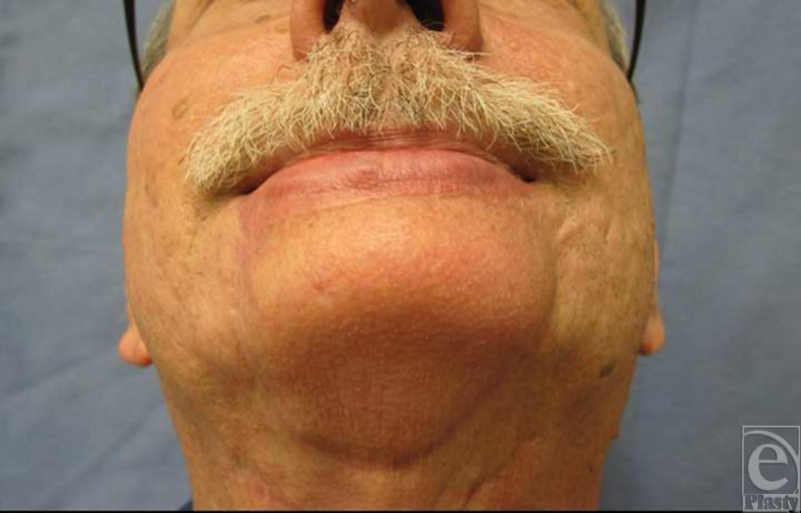
One-year postoperative result of extended chin reconstruction with submental artery island flap—worm's eye view.

After undergoing postoperative radiation therapy with 5040 cGy, the patient complained of drooling and oral incompetence at rest. A static sling composed of autologous tendon tunneled subcutaneously and secured to the malar prominence resulted in normal oral competence and an excellent aesthetic outcome (Figs 4 and 5).

## DISCUSSION

Eccrine carcinomas arise from the intraepithelial ductal portion of the eccrine sweat glands and are rare lesions representing only 0.005% of epithelial cutaneous neoplasms.^7^^-^10 The average age of patient presenting with eccrine carcinoma is 67.5 years. Unlike benign eccrine poroma lesions, malignant lesions are not correlated with areas of high eccrine sweat gland density, but are mostly found on the lower limbs (55%), head (forehead, cheeks, and scalp) (20%), and upper limbs (12%).

Eccrine carinoma lesions may appear as a nodule, infiltrated or erosive plaque, or as a polypoid growth that is frequently ulcerated. Eccrine carcinomas may develop rapidly, reaching 1 cm to several centimeters within a few months, while others may be slow-growing, only reaching these sizes after years of growth.^7^^-^10 Local recurrent or metastatic disease may be associated with multinodularity, ulceration, and rapid growth. Histologic presentation includes irregularly and regularly shaped vesicular hyperchromatic nuclei and abundant clear cytoplasm with variable glycogen granules. The tendency to locally recur necessitates wide local excision of the primary tumor and pathological confirmation of tumor-free margins.

The chin is an anatomical subunit of the face with aesthetic and functional importance, with interplay of multiple convexities and concavities.^1^^-^4,11 The vascular anatomy of the chin arises from the submental branch of the facial artery and local perforating arteries.^12^ The presence of multiple convexities on the chin makes it a challenging subunit to reconstruct.^1^^-^4,13 Coverage of chin defects requires consideration of contour, sensation, expression, normal function, and aesthetic parameters.

Several techniques, including skin grafts, regional flaps, and free tissue transfer, can be utilized in the restoration of maxillofacial defects.^4^,^14^^-^19 The reconstructive tissue chosen should be reliable, restore functionality, be cosmetically acceptable, and provide minimal donor site morbidity while maintaining similar color, texture, size, and thickness of recipient site. Skin grafts typically fail to correct contour deformities and are not recommended in facial reconstruction if other reconstructive methods are available.^3^,^15^ Free tissue transfer, such as the radial forearm flap, are often poor aesthetic matches and involve increased surgical complexity and potential for flap failure associated with microsurgery.^4^,^16^^-^19 Local flaps may yield better aesthetic results than free tissue transfers. As cervical skin meets the conditions of flap suitability, with similar contour, color, and tissue texture of the face, previous authors have attempted the use of flaps from the neck as local pedicled flaps for lower face reconstruction.^4^,^18^^-^20 Five major arteries give rise to the vascular supply of the anterior neck—the subclavian artery, the transverse superficial facial artery, the superior thyroid artery, direct branches from the facial artery, and the submental artery. Platysmal, supraclavicular, infrahyoid and random pattern flaps, including bilobed and rhomboid flaps, as well as lateral V-Y advancements, are some of the local flap techniques for the reconstruction of soft tissue defects of the lower face. These flap techniques, however, are noted for poor donor site scar or mobility resulting in limited success, particularly when used to cover large defects such as the chin subunit.^3^,^4^,^15^,^18^,^20^

There are multiple benefits of the submental artery island flap as a local flap in extended chin reconstruction. These benefits include suppleness of tissue, excellent color and texture match to facial skin, a wide arc of rotation and prominent blood supply, and a well-hidden donor site scar that can be closed primarily due to skin laxity, especially in the aged neck, decreasing submental and neck rhytides.^1^^-^6,14,17,19,21 In addition, the hair-bearing nature of the submentum makes this an ideal flap when transferred to areas of beard growth in males.^13^ The flap can be used in lower face reconstruction without compromise to the lip or oral-buccal continence, and it allows for the convexities and concavities of the menton to be represented with minimal scarring, leaving the patient with an improved aesthetic outcome.^1^^-^4 It is particularly advantageous in older, obese patients for improving the contour of the submental region, but has been used in younger patients without donor site difficulties.^4^,^15^,^19^

The submental artery island flap involves a relatively simple dissection, decreasing operative time, and avoiding microsurgical free tissue transfer.^5^,^6^,^14^,^16^^-^19 This flap has a reliable long vascular pedicle, with the submental artery as its prominent blood supply running above, through, or deep to the anterior belly of the digastric muscle, terminating near the mandibular symphysis. 4^-^6,14,17^-^20 The vein of the submental flap drains into the facial vein. Doppler ultrasound is useful in identifying submental vessels.^3^ Nerve stimulators and careful dissection decrease nerve injury and help preserve functional muscle of surrounding tissue by ensuring the marginal mandibular or cervical branches of the facial nerve remain intact.^4^,^18^^-^20

Disadvantages of the submental flap in chin reconstruction can include a difference in texture of the adipose tissue necessitating debulking and possible damage of the marginal mandibular nerve during the harvesting of the flap. The flap cannot be used when cervical dissection is needed, because lymphatic tissue may be included, resulting in potential for transfer of malignant cells into the reconstructed site.^4^,^18^

The submental artery island flap is a reliable and versatile flap. This flap is an excellent choice for the reconstruction of substantial chin defects. The submental artery flap was chosen for the extended chin reconstruction presented in this case, because it produces good aesthetic donor site appearance, with minimal complications due to its ease of dissection, prominent blood flow, and proximity to the recipient site.

## Acknowledgments

The authors thank Wyatt Payne, MD, Division of Plastic Surgery, University of South Florida, for his efforts in reviewing this paper, and Timothy Westmorland, Medical Media, Bay Pines VA Healthcare System; Michael Harrington, MD, Division of Plastic Surgery, University of South Florida; and Jeffery Cone, MD, Division of Plastic Surgery, University of South Florida, for their help and expertise with figures used in this article.
